# Physical Therapy Interventions in Children With Cerebral Palsy: A Systematic Review

**DOI:** 10.7759/cureus.43846

**Published:** 2023-08-21

**Authors:** Natalie A Gonzalez, Raghavendra R Sanivarapu, Usama Osman, Abishek Latha Kumar, Aishwarya Sadagopan, Anas Mahmoud, Maha Begg, Mawada Tarhuni, Monique N. Fotso, Safeera Khan

**Affiliations:** 1 Pediatrics, California Institute of Behavioral Neurosciences & Psychology, Fairfield, USA; 2 Pulmonary and Critical Care Medicine, California Institute of Behavioral Neurosciences & Psychology, Fairfield, USA; 3 Pulmonary and Critical Care Medicine, Nassau University Medical Center, East Meadow, USA; 4 Internal Medicine, California Institute of Behavioral Neurosciences & Psychology, Fairfield, USA; 5 Geriatrics, Michigan State University College of Human Medicine, East Lansing, USA; 6 Internal medicine, pediatrics, California Institute of Behavioral Neurosciences & Psychology, Fairfield, USA; 7 Obstetrics and Gynecology, California Institute of Behavioral Neurosciences & Psychology, Fairfield, USA

**Keywords:** physiotherapy, exercise training, physical and rehabilitation medicine, physical therapy, cerebral palsy

## Abstract

Cerebral palsy is a group of disorders affecting individuals already from birth. It enormously impacts an individual's physical and emotional life and can bring many challenges to the individual, caregivers, and families. Therefore, it is crucial to investigate interventions that could improve various symptoms in children with cerebral palsy. Our systematic review intends to assess the effect of different exercise and physical therapy interventions in children with cerebral palsy. We used three databases for our article search: PubMed, Medical Literature Analysis and Retrieval System Online (MEDLINE), and PubMed Central (PMC). The combined number of papers found in all databases was 65,412. We then applied our inclusion and exclusion criteria, filters, key terms, and Medical Subheadings (MeSH). After applying our quality assessment tools, we included nine papers in our systematic review. The studies included in our review used various interventions to assess for improvement in symptoms in individuals with cerebral palsy. Interventions included stretching and resistance exercises, horse riding, biking, core stability exercises, slackline training, a home exercise program using an online tool, sit-to-stand exercise program, and functional training. Many studies have shown that interventions improved symptoms like balance, coordination, gait, and cardiovascular endurance in cerebral palsy. This review suggests that some of the included interventions have great potential to improve the symptoms of cerebral palsy and, therefore, can be a great addition to existing training and rehabilitation programs. Given that studies included a relatively small number of participants and were conducted over a short time, more research with a more significant number of participants over a longer time is necessary.

## Introduction and background

Cerebral palsy includes movement disorders affecting two to three out of 1,000 live births. There are various categories of cerebral palsy, including spasticity (the most common), dyskinesia, ataxia, or a mixed type. Due to brain injury in cerebral palsy, different aspects of an individual's life, including movement, balance, and gait, are affected [1,2]. Many risk factors for cerebral palsy were identified over time. During the preconception period, potential risk factors include systemic diseases in the mother as well as drug abuse, malnourishment, infections, and disorders of the immune system [1,3-8]. During the prenatal period, risk factors include but are not limited to oligohydramnios, polyhydramnios, infection in pregnancy, placental diseases, intrauterine growth restriction, twin gestations, or premature rupture of membranes [1,3-8]. The majority of the etiology in cerebral palsy is affecting the perinatal period. Several risk factors were identified, including preterm birth, meconium aspiration syndrome, prolonged labor, cesarean section, and asphyxia [1,3-8]. Less than 10% result from hypoxia during labor. However, it is often impossible to identify an attributable cause [1]. Cerebral palsy is diagnosed clinically, but imaging techniques like magnetic resonance imaging (MRI) can be used to gather further information [9]. A cohort study was conducted to evaluate the neuroimaging characteristic of infants with cerebral palsy. In this study, arterial infarction was found in 22% of the infants. In addition, brain malformation was found in 14% of the infants, and in 12% of the infants, abnormalities of the periventricular white matter were observed [10].

The severity of the symptoms of cerebral palsy can range from mild to severe. Therefore, much of the burden last on the caregivers as children with cerebral palsy often depend entirely on them [9]. Cerebral palsy can result in various symptoms, including balance issues, limited range of motion, equinus deformity, reduced hand function, sensory deficits, and hip disorders. To measure the severity of symptoms and also to monitor improvement after therapy interventions, the most commonly used tool is the Gross Motor Function Classification System (GMFCS) [1].

Individuals with cerebral palsy are commonly affected by other health-related issues, including speech and hearing impairments, seizures, cognitive dysfunction, osteoporosis, and behavioral problems. It can also cause hip displacement or sleep disorders [1]. Cerebral palsy can also affect the quality of life in an individual as well as the dynamics in the family. A study showed that the quality of life in children with cerebral palsy is reduced compared to unaffected children [11]. Caregivers of children with cerebral palsy are more likely to experience a higher level of stress compared to caregivers of children without the disease. It was also found that caregivers are more likely to be anxious, depressed, and more dissatisfied with their lives [12,13,14]. Especially epilepsy is highly prevalent in individuals with cerebral palsy, with some studies reporting incidences from 15% to 55%-60% or even up to 90%-94%. The combination of cerebral palsy and epilepsy places a child at a higher risk for sudden unexpected death in epilepsy (SUDEP) [9].

A study evaluated the effects of antenatal magnesium sulfate in premature infants. Findings suggested that this intervention has a neuroprotective effect on premature infants and can lower the risk of cerebral palsy [15]. To promote lung maturation, premature infants often receive antenatal steroids. Studies suggested that this could potentially lead to a higher risk for cerebral palsy. However, the effect of antenatal steroids on neurodevelopmental impairment is not fully clear and requires further studies [16,17].

The treatment for cerebral palsy includes multiple specialties, including physicians, surgical specialists, social workers, educators, psychologists, physical and occupational therapists, and speech-language pathologists. The treatment is based on the individual's symptoms and involves a multidisciplinary team [1]. Various interventions exist intending to reduce the symptoms of cerebral palsy, including muscle relaxants, intramuscular botulinum toxin, and surgical intervention such as a selective dorsal rhizotomy. Physical and occupational therapies are significant aspects of training interventions [1,18,19,20]. There was a time when it was falsely assumed that strengthening exercises could worsen spasticity and abnormal movements. However, studies did show that resistance training can positively enhance gait, balance, and motor function in individuals with cerebral palsy [21].

We wrote this systematic review to assess the effect of different exercise and physical therapy interventions in children with cerebral palsy. Our paper aims to focus on the most current data regarding other exercise programs and physical therapy interventions in children with cerebral palsy and if those interventions can improve various symptoms, including but not limited to balance, gait, and coordination.

## Review

Methods 

**Figure 1:**
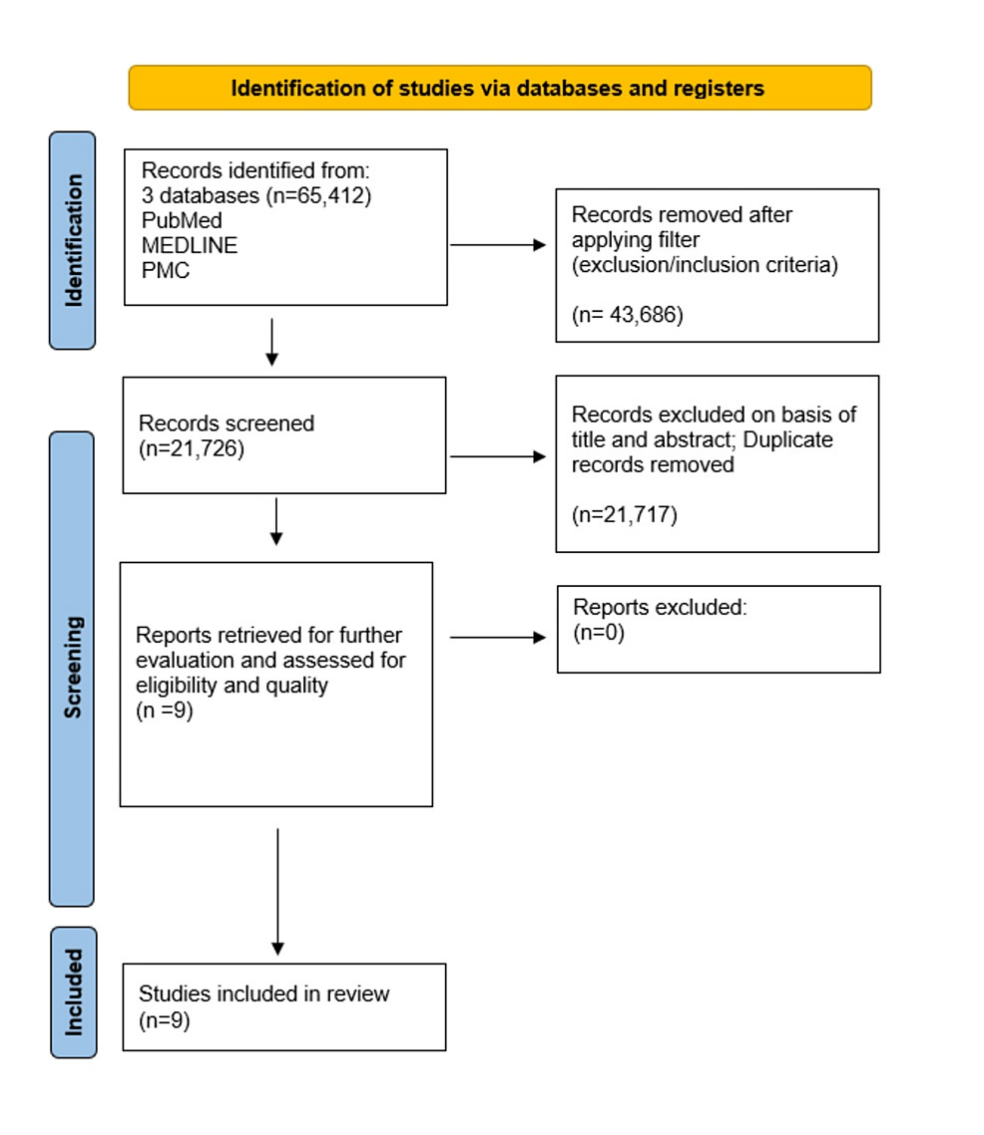
PRISMA flowchart of selected articles. PRISMA, Preferred Reporting Items for Systematic Reviews and Meta-Analyses; MEDLINE, Medical Literature Analysis and Retrieval System Online; PMC, PubMed Central

We followed the Preferred Reporting Items for Systematic Reviews and Meta-Analyses (PRISMA) Guidelines 2020 for this systematic review [22]. To give a more detailed illustration of our article selection, we used the PRISMA flowchart (Figure 1) [22].

Search Strategy

We used the following databases for our paper: PubMed, Medical Literature Analysis and Retrieval System Online (MEDLINE), and PubMed Central (PMC). We created a Medical Subject Heading (MeSH) concept with the key terms "Cerebral Palsy," "Physical therapy," and "Physical and rehabilitation medicine." We decided to use the following key terms "Children," "Cerebral Palsy," "Physical Therapy," "Exercise Training," Movement Therapy," and "Physiotherapy." We used different combinations of these key terms for our three databases. Given the significant number of papers we found, we used our inclusion and exclusion criteria to reduce the number of papers. We screened the articles based on their title and abstract, and finally, we used different quality checks to finalize our selection. In the end, we selected nine papers for our systematic review.

Inclusion Criteria

We decided to use only articles in English published within the last five years. On April 18, 2023, we completed our database search. All our final selected papers were open-access.

Exclusion Criteria

Our exclusion criteria were articles in another language than English with more than five years of publication date.

Results

Initially, we found 65,412 in the databases PubMed, MEDLINE, and PMC. We removed 43,686 articles after applying our exclusion and inclusion filter to each database. We screened the remaining 21,726 articles and removed 21,717 based on their titles, abstracts, or duplicates. We assessed nine papers for quality, all of which satisfied our criteria. We used all nine articles in our systematic review. Table 1 gives a more detailed description of our selected papers.

**Table 1:** Selected articles evaluating different physical interventions in children with cerebral palsy. LIFT, lower extremity functional training; 1MWT, 1-minute walk test; ABILOCO-Kids, a questionnaire used to evaluate locomotory ability; AVr-Ex, accommodating variable-resistance exercise program; PRE, progressive resistance exercise; Borg-RPE, Borg scale rate of perceived exertion; GMFM, Gross Motor Function Measure; GMFCS, Gross Motor Function Classification System; WeeFIM, Functional Independence Measure for Children; FTSST, Five Times Sit-to-Stand Test; MCSI, Modified Caregiver Strain Index

Study	Publication year	Location	Type of study	Total patient population	Outcome
Surana et al. [23]	2019	United States	Randomized controlled trial	40 participants, aged between two and 13 years	This study used LIFT as an intervention. The intervention group showed improvement in the 1MWT and in the ABILOCO-Kids. Other measures included a 30-second chair rise test, single-leg stance, and walking speeds, which did not differ between the intervention and control groups.
Elnaggar et al. [21]	2021	Saudi Arabia	Randomized controlled trial	36 participants, aged between eight and 16 years	This study used the AVr-Ex program to evaluate balance and improvement in symmetric gait in children. Using this exercise intervention on top of established physical rehabilitation training showed successful progress in the intervention group compared to the nonintervention group.
Fosdahl et al. [24]	2019	Norway	Randomized controlled trial	37 participants, aged between seven and 15 years	The intervention group underwent a combination of stretching and a PRE program. The study measured the outcome with gait parameters. However, after completing the program, the intervention group had no significant changes in their gait parameters compared to the control group.
Elshafey et al. [25]	2022	Egypt	Randomized controlled trial	45 participants, aged between five and nine years old	A core stability exercise program was used as an intervention to determine its effect on coordination and balance in children with cerebellar ataxic cerebral palsy. The study's outcome showed that if the exercise program is combined with a physical therapy program, balance and coordination could be improved in the intervention group.
Hjalmarsson et al. [26]	2020	Sweden	Clinical trial	15 participants, aged between nine and 29 years	This study used RaceRunning training, a three-wheeled running bike suitable for individuals with cerebral palsy. Participants underwent this intense exercise program over 12 weeks. Results showed that individuals improved their cardiorespiratory endurance. Other effects included an increase in passive hip flexion, a decrease in ankle dorsiflexion, and an increase in thickness in the medial gastrocnemius muscle. The average and maximum heart rate and Borg-RPE measures were unchanged before and after the exercise program.
González et al. [27]	2020	Spain	Randomized controlled trial	27 participants, aged between nine and 16 years	This study used slackline training to assess postural control in children with spastic cerebral palsy. Participants in the intervention group were able to improve their postural control as well as their motor skills compared to the control group. In addition, the slackline training was reported to not feel too exhaustive for the participants.
Johnson et al. [28]	2020	Australia	Randomized controlled trial	54 participants, aged between six and 17 years	The intervention group used a home exercise program via the online tool Physitrack, while the control group used paper-based methods. None of the groups was superior in terms of number of exercises used. There was no significant difference in setting goals, exercise performance, or subjective joy.
Zaliene et al. [29]	2018	Lithuania	Clinical trial	15 participants, aged between three and 19 years	GMFM and GMFCS were used to assess the effects of riding on the mobility of individuals with cerebral palsy. Group 1 included advanced riders, whereas beginners were in Group 2. The beginner group had no significant improvement in their mobility. However, half of the advanced riders improved their gross motor functions considerably.
Chaovalit et al. [30]	2021	Thailand	Randomized controlled trial	38 participants, aged between two and 12 years	This study used a sit-to-stand exercise program to assess self-care and mobility in children with cerebral palsy. The intervention group used the sit-to-stand exercise with physiotherapy, whereas the control group only used routine physiotherapy. The outcomes were measured using WeeFIM, FTSST, and MCSI. The intervention group improved self-care and mobility compared to the control group.

Quality Check

We used various quality appraisal tools to evaluate the quality of our selected papers. Most of our selected papers were randomized controlled trials. Therefore, we used the Cochrane Risk of Bias Tool. For nonrandomized clinical trials, we chose the Newcastle-Ottawa Tool Scale.

Discussion

Randomized Controlled Trials Assessing the Effectivity of Physical Intervention in Children With Cerebral Palsy

Surana et al. conducted a randomized controlled trial to assess the effect of lower extremity intensive functional training (LIFT) on gait and gross motor skills in children with unilateral spastic cerebral palsy [23]. The trial included 40 participants aged between two and 13 years. The intervention group used LIFT for 90 hours, and the control group used Hand-Arm Bimanual Intensive Therapy (H-HABIT) over nine weeks. The intervention LIFT included different exercises, focusing on building resistance, coordination, and balance. Individuals completed the intervention in their homes [23]. Researchers assessed the outcome with the one-minute walk test (1MWT). The intervention group had better 1MWT results than the control group. Researchers used the 10-Meter Walk Test, 30-second chair rise, single-leg stance, and ABILOCO-Kids to measure further results. Besides the ABILOCO-Kids, a questionnaire used to evaluate locomotory ability, no significant difference was measured between the intervention and control group [23]. Overall, using LIFT as an intervention is a promising way to improve gross motor skills and gait in children with cerebral palsy. The study suggests that more studies with different amounts of training hours, another training schedule, or focusing on personal goals from the individuals are necessary to evaluate the effectiveness of the intervention [23].

The randomized controlled trial from Elnaggar et al. used the variable-resistance exercise (AVr-Ex) program to evaluate its effect on balance and symmetric gait in children with hemiparetic cerebral palsy [21]. This trial included 36 participants aged between eight and 16 years. The intervention group used the AVr-Ex program with a physical therapy program, whereas the control group only performed a regular physical therapy program. Both groups completed the training over eight weeks, with three sessions per week. A pediatric physical therapist monitored all sessions [21]. Researchers measured the effects of the training before and after each session. The trial used different measures, including weight-bearing symmetry, gait symmetry, and dynamic balance. The intervention group showed improvement in all outcome measures compared to the control group, suggesting that AVr-Ex, on top of conventional physical rehabilitation, can be an effective way to improve gait and balance in children with cerebral palsy. The study suggests that more research assessing the effect of the AVr-Ex program over a more extended period is necessary to investigate the benefits better [21].

Fosdahl et al. conducted a randomized controlled trial to evaluate the effect of combined strength and stretching training on gait function in children with cerebral palsy [24]. The study included 37 participants aged between seven and 15 years. Researchers conducted this trial over 32 weeks. The intervention group completed a 16-week program with a combination of hamstring stretching exercises with progressive resistance exercise (PRE) three times a week, with an additional 16 weeks with maintenance exercises conducted once weekly. The control group underwent their usual care, with their physiotherapist instructed not to try any new treatment forms during the study [24]. The outcomes were measured with kinematic gait variables, gait speed, step length, Gait Deviation Index (GDI), and the six-minute walk test (6MWT). Both groups showed improvement with the 6MWT, but the study did not find any significant difference with all other measures. The study suggests that more research in the future should include more gait-specific exercises to evaluate for improvement in gait function [24].

The randomized controlled trial from Elshafney et al. assessed the use of a core stability exercise program on balance and coordination in children with cerebellar ataxic cerebral palsy [25]. The trial included 45 individuals aged between five and nine years. The intervention group used core stability exercises and physical therapy; the control group underwent only a physical therapy program. The program was completed thrice weekly over two months [25]. The outcome was measured before the start of the program and after completion. The researchers used the Scale for the Assessment and Rating of Ataxia (SARA), the Bruininks-Oseretsky Test of Motor Proficiency (BOT-2), the Balance Error Scoring Systems Scale (BESS), and the HUMAC Balance System to measure improvement in balance and coordination [25]. The intervention group improved balance, coordination, and ataxia more than the control group. The study suggests that more research in this field is necessary to evaluate the effectiveness of a core exercise program in the long term and the effect on more functional activity [25].

González et al. conducted a randomized controlled trial to assess the effectiveness of slackline training on postural control in children with spastic cerebral palsy [27]. This study included 27 participants aged between nine and 16 years. The study was conducted over six weeks, with both groups receiving their usual care and the intervention group completing additional three slackline training sessions per week. The outcomes were measured using the center-of-pressure (COP) parameters, countermovement jump test, and Abalakov test [27]. Results showed that the group members who completed the slackline training was able to improve their postural control as well as their motor skills in comparison to the control group. Individuals did not feel that the slackline training was too exhausting. Therefore, the study suggests this exercise could effectively add to the existing training without causing too much fatigue in children with spastic cerebral palsy. Further, the study suggests that future studies should include a more significant number of participants and more training interventions that focus on balance with slackline training [27].

Johnson et al. conducted a randomized controlled trial to evaluate exercise adherence and performance and accomplishing goals using an online exercise prescription tool named Physitrack in children with disabilities, including cerebral palsy [28]. The study included a total number of 54 individuals aged six to 17 years. The study duration was eight weeks, with both groups completing exercises at home. The intervention group received all the information regarding their activities via the online tool Physitrack. Physitrack can be used on the website or as an application on the phone and also demonstrates videos of the exercises. The control group completed the exercises using a paper-based method [28]. The results were measured using the Canadian Occupational Performance Measure (COPM), Correctness of Exercise Performance (COEP), System Usability Scale (SUS), and Physical Activity Enjoyment Scale (PACES). The intervention and control groups both reported an enhancement in their goal activities. The researchers did not report a significant outcome in terms of goal achievement, performance of exercises, or subjective joy. The researchers suggest that more studies with more participants are necessary to assess the effectiveness of online exercise tools [28].

Chaovalit et al. conducted a randomized controlled trial to assess the effect of a sit-to-stand exercise program on self-care and mobility in children with cerebral palsy [30]. The study included 38 participants aged two to 12 years and was conducted over six weeks. The intervention group used sit-to-stand exercises in combination with conventional physiotherapy, and the control group used physiotherapy alone. Routine physiotherapy, as well as sit-to-stand training, were done five times a week [30]. A physiotherapist observed two out of five sessions, and the caregivers supervised three sessions. The outcomes were measured using the Functional Independence Measure for Children (WeeFIM), Five Times Sit-to-Stand Test (FTSST), and Modified Caregiver Strain Index (MCSI). Self-care and mobility improved more in the intervention group than in the control group. The study suggests this exercise could be valuable to therapies for children with cerebral palsy [30].

Clinical Trials Evaluating Physical Intervention in Children With Cerebral Palsy

Hjalmarsson et al. conducted a clinical trial to study the effects of RaceRunning training in individuals with cerebral palsy [26]. In the trial, 15 individuals participated, aged nine to 29 years. Researchers conducted this clinical trial over 12 weeks, with two training sessions per week. RaceRunning is a three-wheeled running bike made suitable for individuals with cerebral palsy. Researchers measured the effects of the intervention before starting the program, after four weeks, after eight weeks, and after 12 weeks. The outcome was assessed using 6-minute RaceRunning test (6-MRT), passive range of motion (pROM) of hip, knee and ankle, perceived exertion using the Borg scale (Borg-RPE), average and maximum heart rate, and the muscle thickness of the thigh and lower leg using ultrasound [26]. The study highlights that RaceRunning contributed to improving cardiorespiratory endurance in participants. Results showed that individuals had an increase in passive hip flexion, a decrease in ankle dorsiflexion, and an increase in skeletal muscle thickness. Heart rates and Borg-RPE measures were the same before and after the completion of the study. Given the study's results, the researchers suggest that RaceRunning can effectively train individuals with cerebral palsy [26].

A clinical trial from Zaliene et al. evaluated possible improvements in the mobility of individuals with cerebral palsy using horse riding [29]. The clinical trial included 15 individuals aged between three and 19 years. The first group had advanced riders, and the second group consisted of beginners. The advanced riders completed one weekly riding session throughout one to four years, and the beginners completed 10 riding sessions within 10 weeks. The outcome was measured using Gross Motor Function Measure (GMFM) and Gross Motor Function Classification System for CP (GMFCS). The advanced group was assessed before and after the study. The effects of the riding sessions in the beginner group were measured before, during, and after the training [29]. There was no significant difference before and after the intervention in the beginner group. Half of the advanced riders significantly improved their gross motor function. Limitations of the study were the different times and frequencies between the riding sessions of both groups. Researchers noted that the beginner group had no other rehabilitation training at the time of the study. In contrast, the advanced group received rehabilitation services over the study period [29]. 

Limitations

This systematic review focused on the most current data within the last five years. However, that could also be a limitation as we did not include papers published before that. Another limitation is that we decided only to use papers in the English language. Finally, there were limited articles on this topic; therefore, more in-depth research would be needed.

## Conclusions

Our systematic review aimed to assess the effect of physical interventions in children with cerebral palsy, including physical therapy and training exercises. Most of our included studies reported improvement in various symptoms in individuals with cerebral palsy, including but not limited to gait, balance, coordination, and cardiorespiratory endurance. Some limitations of the studies included were the number of participants and the study duration. We recommend that future studies involve a larger number of participants and span an extended duration. Given the tremendous effect of cerebral palsy on individuals and their caregivers, more research in this field must be conducted to assess interventions that could positively influence symptoms in children with cerebral palsy and improve their quality of life.
